# Intra-Aortic Balloon Pump During Percutaneous Coronary Intervention in ST-Elevation Myocardial Infarction With High Thrombus Burden and Cardiogenic Shock

**DOI:** 10.7759/cureus.34188

**Published:** 2023-01-25

**Authors:** Rajeev Chauhan, Parminder S Otaal

**Affiliations:** 1 Department of Cardiology, Post Graduate Institute of Medicine and Research, Chandigarh, IND; 2 Department of Cardiology/Interventional Cardiology, Post Graduate Institute of Medical Education and Research, Chandigarh, IND

**Keywords:** lv assist, high risk pci, high thrombus burden, st elevation myocardial infarction (stemi), intra-aortic balloon pump (iabp)

## Abstract

Background: Percutaneous coronary intervention (PCI) in ST-elevation myocardial infarction (STEMI) with high-grade thrombus is a high-risk intervention associated with poor clinical outcomes. Circulatory support with an intra-aortic balloon pump (IABP) during PCI may potentially improve coronary hemodynamics and clinical outcomes in such patients. As existing data on this situation are sparse, we did an observational study to determine short-term outcomes of PCI with IABP support in STEMI patients with high thrombus burden.

Objectives: To determine whether IABP has a potential role in improving outcomes in patients with STEMI with high thrombus burden who are undergoing PCI.

Methods and results: Thirty consecutive patients of STEMI with high thrombus burden undergoing PCI with IABP assistance were included. Ninety-three percent of patients had a cardiogenic shock. Clinical and angiographic outcomes assessed include a change in left ventricular ejection fraction (LVEF), 30-day mortality, and assessment of TIMI (thrombolysis in myocardial infarction) flow, TIMI frame count, and TIMI myocardial perfusion grade in the culprit vessel. IABP was initiated before coronary angiography in 36.6% (n=11), between angiography and PCI in 30% (n=9), and after PCI in 33.3% (n=10) of patients. During the 30-day follow-up period, 50% (n=15) of patients died. 86.6% (n=13) of survivors had pre-PCI IABP initiation compared to only 46.6% (n=7) among those who died (p=0.020). With pre-PCI IABP initiation (n=20), 30-day mortality was 35% (n=7) compared to 80% (n=8) with post-PCI IABP initiation (n=10) (p=0.020).

Conclusion: IABP initiation before PCI in STEMI complicated by cardiogenic shock and high angiographic thrombus burden can decrease mortality without any effect on angiographic parameters.

## Introduction

Coronary artery disease (CAD) is the single most important cause of mortality and loss of disability-adjusted life years (DALYs) worldwide [1]. One of the most serious presentations of CAD is ST-elevation myocardial infarction (STEMI), in which the mortality rate remains considerable, varying from 4% to 12% and it approaches 40-50% in those with associated cardiogenic shock [2]. Angiographic predictors of adverse outcomes in STEMI include absence of collaterals, type of culprit vessel, proximal location of lesion, low TIMI (thrombolysis in myocardial infarction) flow in culprit vessel, and higher thrombus burden [3]. High thrombus burden, a significant predictor of major adverse cardiovascular events and mortality, has also been associated with angiographic complications like no reflow, microvascular hypoperfusion, and periprocedural myocardial injury [4]. Pre-PCI (percutaneous coronary intervention) assessment for high thrombus burden in STEMI patients provides a window of opportunity to use adjunctive therapies like mechanical circulatory support (MCS) device at the earliest if a large amount of myocardium is at risk or presence of significant thrombus or in patients with impaired hemodynamics [5]. Intra-aortic balloon pump (IABP) can improve coronary blood flow and diminish the workload of the ischemic myocardium during high-risk PCI [6]. Despite the beneficial effects of prophylactic IABP during PCI in some studies, IABP-SHOCK II Trial showed no survival advantage for IABP use in STEMI with cardiogenic shock [7-9]. Surprisingly, most (86.6%) patients in this trial received IABP after the revascularization leaving an enigma whether earlier initiation of IABP could have affected the results [10].

Despite confounding evidence in its favor, 2011 ACC (American College of Cardiology)/AHA (American Heart Association) guidelines on PCI give Class IIb recommendation for use of MCS device as an adjunct to PCI in high-risk patients such as those with severely depressed EF undergoing PCI and/or those with cardiogenic shock [11]. Due to the limited availability of MCS devices apart from IABP in resource-poor countries and IABP being more cost-effective than Impella 2.5 with a similar mortality rate and safety profile, it might play an important role in providing favorable outcomes in high-risk situations [12]. Despite the abundance of data on the role of IABP during PCI in a variety of clinical settings, it has not been specifically evaluated in STEMI complicated by cardiogenic shock with high-grade angiographic thrombus.

Given the abovementioned facts and controversies surrounding IABP, we conducted a prospective observational study to assess outcomes of IABP use in patients undergoing PCI for STEMI with high thrombus burden at our tertiary care teaching institute.

## Materials and methods

This is a prospective observational study conducted at one of the largest tertiary care centers in a developing nation conducted from September 2018 to December 2019.

Inclusion criteria

The study included 30 consecutive patients aged >18 years presenting with STEMI associated with high angiographic thrombus burden (Grades 4 and 5) undergoing primary or rescue PCI with IABP support. TIMI study group has defined thrombus grade as per classification by Gibson et al. [13]. Specifically, a Grade 4 thrombus was defined as a definite large thrombus with a dimension >2 vessel diameter and Grade 5 as definite complete thrombotic occlusion of a vessel. Thrombus grade 4 and 5 has been defined as high-grade thrombus [14]. Primary PCI is defined as an emergent percutaneous catheter intervention in the clinical setting of STEMI, without previous fibrinolytic treatment. PCI after failed thrombolysis for patients with continuing or recurrent myocardial ischemia is defined as rescue PCI.

Exclusion criteria

The exclusion criteria were as follows:

a. severe aortic regurgitation;

b. aortic dissection;

c. severe coagulopathy;

d. severe sepsis;

e. patients who underwent emergency coronary artery bypass grafting.

Ethical clearance

The study protocol conforms to the ethical guidelines of the Declaration of Helsinki and was reviewed and approved by the institutional ethical committee.

Patient care and management

All 30 patients underwent PCI with IABP support. Ninety-three percent of patients were in cardiogenic shock. Cardiogenic shock was defined as the patient having systolic blood pressure (SBP) <90 mm Hg for 30 min or inotropes use to maintain SBP >90 mm Hg along with evidence of end‐organ hypoperfusion or increased filling pressures [15]. Every patient underwent a detailed clinical evaluation including history, physical examination, electrocardiogram, chest x-ray, and echocardiography. Patients were managed as per standard protocols. The timing of IABP initiation was at the discretion of the primary operator. Sub-group analysis regarding the timing of IABP initiation and outcomes was also done. All patients were assessed for clinical and angiographic outcomes. Thereafter they were followed up for 30 days.

Outcome parameters

The clinical and angiographic parameters were used to assess outcomes as mentioned in Table 1.

**Table 1:** Outcome parameters (clinical and angiographic parameters) LVEF, left ventricular ejection fraction; TIMI, "Thrombolysis in Myocardial Infarction" study group; cTFC, corrected TIMI frame count; TMPG, TIMI myocardial perfusion grade; PCI, percutaneous coronary intervention.

S No	Parameter	Variable
1	Clinical parameters	30-day all-cause mortality
		LVEF at pre-discharge and at 30 days
2	Angiographic parameters (pre- and post-PCI) in culprit vessel	Assessment of TIMI flow
		Assessment of cTFC
		Assessment of TMPG

Assessment for coronary flow in the culprit vessel was done by the TIMI flow-grading system [16]. Objective assessment of coronary flow was done by using corrected TIMI frame count (cTFC), in which the number of cine-frames required for the dye to reach standardized distal landmarks are counted [17]. Assessment for TIMI myocardial perfusion grade (TMPG) was carried out as per the standard angiographic method used by Gibson et al. [13].

Statistical analysis

The parameters were assessed as per standard statistical methods. All quantitative or continuous variables were estimated using measures of central tendency (mean, median) and measures of central dispersion (standard deviation). Qualitative or categorical variables were described as frequencies and proportions. For group comparisons of categorical variables, the chi-square test or Fisher's exact test was used. For normally distributed data, a student's t-test was used. A p-value of less than 0.05 was considered significant. For the comparison of more than two variables, the analysis of variance (ANOVA) test was used.

Ethical approval

The institution's ethics committee approved the study as per PGIMER letter IEC/2019/258 dated 11 February 2019.

## Results

Baseline clinical characteristics

The baseline clinical characteristics of the study population are given in Table 2.

**Table 2:** Baseline characteristics of the study population SD, standard deviation; PCI, percutaneous coronary intervention; CABG, coronary artery bypass grafting; MI, myocardial infarction; LVEF, left ventricular ejection fraction.

S No	Baseline clinical characteristics	Frequency
1	Age, years±SD	60.46±10.6
2	Males, n (%)	25 (83.3%)
3	Females, n (%)	5 (16.6%)
4	Diabetes, n (%)	12 (40%)
5	Hypertension, n (%)	21 (70%)
6	Smokers, n (%)	18 (60%)
7	Past history of PCI, n (%)	4 (13.3%)
8	Post-CABG, n (%)	1 (3.3%)
9	Anterior wall MI, n (%)	19 (63.3%)
10	Inferior wall MI, n (%)	11 (36.6%)
11	Thrombolysis, n (%)	10 (33.3%)
12	Pre-procedure LVEF (%±SD)	34±8.13%
13	Patients with pre-procedure LVEF ≤35%, n (%)	16 (53.3%)
14	Cardiogenic shock, n (%)	28 (93.3%)
(a)	At presentation, n (%)	10 (33.3%)
(b)	Prior to procedure, n (%)	8 (26.6%)
(c)	During procedure, n (%)	10 (33.3%)
15	Left ventricular failure, n (%)	6 (20%)
16	Complete heart block, n (%)	5 (16.6%)

The total number of patients included in the study was 30. The average age of the study population was 60.46±10.6 years with 83.3% males. 93.3% of patients (n=28) had a cardiogenic shock, of which 33.3% (n=10) had a shock at admission, eight patients (n=26.6%) developed shock prior to angiography whereas 10 patients (33.3%) developed shock during/immediately after the procedure (angiography/PCI). Four patients had undergone PCI in the past for chronic stable angina and they had normal left ventricular function during a previous follow-up visit. During the index procedure, two patients also underwent manual aspiration of thrombus.

Angiographic characteristics

Pre- and post-PCI angiographic characteristics of the study population are given in Table 3.

**Table 3:** Pre-PCI versus post-PCI angiographic characteristics TIMI, "Thrombolysis In Myocardial Infarction" study group; PCI, percutaneous coronary intervention; TMPG, TIMI myocardial perfusion grade. *p < 0.05.

S No	Angiographic feature	Pre-PCI (N=30)	Post-PCI (N=30)	p-Value
1	TIMI thrombus Grade 0 , n (%)	0	14 (46.6%)	-
2	TIMI thrombus Grade 1, 2, and 3, n (%)	0	11 (36.6%)	-
3	TIMI thrombus Grade 4, n (%)	19 (63.3%)	3 (10%)	0.000018*
4	TIMI thrombus Grade 5, n (%)	11 (36.6%)	2 (6.6%)	0.0047*
5	TIMI flow 0/1, n (%)	15 (50%)	6 (20.0%)	0.014*
6	TIMI flow 2/3, n (%)	15 (50%)	24 (80.0%)	0.014*
7	TMPG 0/1, n (%)	15 (50%)	7 (23.3%)	0.032*
8	TMPG 2/3, n (%)	15 (50%)	23 (76.6%)	0.032*

Pre-PCI

Culprit vessel TIMI flow was 0 in 9 (34.61%), 1 in 6 (20%), and 2 in the rest of the 15 (50%) cases. Angiographic myocardial perfusion as assessed by TMPG was grade 0 in 12 (40%), 1 in 3 (10%), and 2 in 15 (50%) patients.

Post-PCI

Post-PCI, culprit vessel TIMI thrombus grade was 0 in 46.6% (n=14), 1 in 23.3% (n=7), 2 in 13.3% (n=4), 4 in 10% (n=3), and Grade 5 in 6.6% (n=2) patients. 16.6% (n=5) patients continued to exhibit high-grade thrombus (Grade 4 or 5) despite PCI. TIMI 3, TIMI 2, TIMI 1, and TIMI 0 flow were seen in 16 (53.3%), 8 (26.6%), 4 (13.3%), and 2(6.6%) patients, respectively. 43.3% (n=13) patients had TMPG 3, 33.3% (n=10) had TMPG 2, 13.3% (n=4) had TMPG 1, and only 10% (n=3) had TMPG 0.

Clinical outcomes

During the 30-day follow-up period, 15 (50%) patients died, of which 14 died during index hospitalization and one patient died one week after discharge due to refractory ventricular tachycardia at a nearby health clinic. Analysis was done to know the factors associated with survival and the effect of the timing of IABP initiation on clinical outcomes.

Subgroup analysis of survivors versus non-survivors

Subgroup analysis of survivors versus those who succumbed by 30 days of follow-up is as shown in Table 4.

**Table 4:** Subgroup analysis between those who survived at 30 days versus those who died SD, standard deviation; AWMI, anterior wall myocardial infarction; IWMI, inferior wall myocardial infarction; PCI, percutaneous coronary intervention; LVEF, left ventricular ejection fraction; IABP, intra-aortic balloon pump; TIMI, "Thrombolysis in Myocardial Infarction" study group. *p < 0.05.

S No	Feature	Survived at 30 days (N=15)	Died within 30 days (N=15)	p-Value
1	Age, years±SD	58.8±10.6	62.13±13.7	0.198
2	Diabetes, n (%)	7 (46.6%)	5 (33.3%)	0.453
3	Hypertension, n (%)	10 (66.6%)	11 (73.3%)	0.689
4	Smoking, n (%)	10 (66.6%)	8 (53.3%)	0.453
5	AWMI, n (%)	7 (46.6%)	12 (80%)	0.058
6	IWMI, n (%)	8 (53.3%)	3 (20%)	0.058
7	Cardiogenic shock, n (%)	14 (93.3%)	14 (93.3%)	1.0
8	Left ventricular failure, n (%)	2 (13.3%)	4 (26.6%)	0.368
9	LVEF ≤ 35%, n (%)	8 (53.3%)	8 (53.3%)	1.0
10	Pre-angiography IABP, n (%)	6 (40%)	5 (33.3%)	0.703
11	Post-angiography pre-PCI IABP, n (%)	7 (46.6%)	2 (13.3%)	0.046*
12	Pre-PCI IABP, n (%)	13 (86.6%)	7 (46.6%)	0.020*
13	Post-PCI IABP, n (%)	2 (13.3%)	8 (53.3%)	0.020*
14	Left main disease, n (%)	1 (6.6%)	4 (26.6%)	0.141
15	Double vessel disease, n (%)	8 (53.3%)	8 (53.3%)	1.0
16	Triple vessel disease, n (%)	4 (26.6%)	4 (26.6%)	1.0
17	Pre-PCI TIMI 0, n (%)	5 (33.3%)	4 (26.6%)	0.689
18	Pre-PCI TIMI 1, n (%)	2 (13.3%)	4 (26.6%)	0.362
19	Post-PCI TIMI 2, n (%)	3 (20%)	5 (33.3%)	0.406
20	Post-PCI TIMI 3, n (%)	12 (80%)	4 (26.6%)	0.003*
21	Post-PCI TIMI 2/3 flow	15 (100%)	9 (59.9%)	0.006*
22	≥1 vessel PCI, n (%)	4 (26.6%)	4 (26.6%)	1.0
23	Acute limb ischemia, n (%)	0	1 (6.6%)	0.307
24	Tamponade, n (%)	0	1 (6.6%)	0.307

Timing of IABP Initiation and Survival

Our results show that survival was significantly better in patients in whom IABP was initiated before PCI. Among survivors (n=15), 13 (86.6%) patients had IABP initiation before PCI compared to only 7 (46.6%) patients among those who eventually succumbed (p=0.020) whereas, among those with post-PCI IABP initiation, the survival rate was significantly lower compared to mortality (13% vs 53%, p=0.020).

TIMI Flow and Survival

We also observed that 80% (n=12) of the survivors had TIMI 3 flow in the culprit vessel after PCI as compared to only 26.6% (n=4) among those who died. The difference was statistically significant (p=0.003). All (100%) of those who survived 30 days (n=15) had TIMI 2 or 3 flow after PCI in the culprit vessel as compared to only 53.3% (n=8) among those who died with a statistically significant (p=0.006) difference.

*Left Ventricular Ejection Fraction* *and Survival*

A statistically significant improvement in left ventricular ejection fraction (LVEF) with a mean increase of 6.67±5.5% at 30 days was observed in patients who survived (p=0.035).

Other variables like age, hypertension, diabetes, left main involvement, and severe left ventricular dysfunction were not statistically different between these two groups.

Thus, our results suggest that factors associated with better short-term survival in patients of STEMI with cardiogenic shock and high thrombus burden include improved TIMI flow rates, improvement in LVEF, and pre-PCI initiation of IABP.

Subgroup analysis based on the timing of IABP initiation

A subgroup analysis to further evaluate the effect of the timing of IABP initiation on various parameters is shown in Table 5.

**Table 5:** Subgroup analysis between pre-angiography IABP, IABP after angiography but before PCI and post-PCI IABP initiation AWMI, anterior wall myocardial infarction; IWMI, inferior wall myocardial infarction; PCI, percutaneous coronary intervention; IABP, intra-aortic balloon pump; TIMI, "Thrombolysis in Myocardial Infarction" study group; LVEF, left ventricular ejection fraction; TMPG, TIMI myocardial perfusion grade; ANOVA, analysis of variance. *p < 0.05, ^Ɨ^ANOVA test.

S No	Variable	Pre-angiography IABP (N=11)	IABP between angiography and PCI (N=9)	Post-PCI IABP (N=10)	p-Value
1	Age (years) ±SD	64±9.72	56.77±12.58	59.9±9.26	0.319^Ɨ^
2	Pre-PCI TIMI flow 0, n (%)	3 (27.2%)	4 (44.4%)	2 (20%)	0.494
3	Pre-PCI TIMI flow 1, n (%)	3 (27.2%)	1 (11.1%)	2 (20%)	0.667
4	Pre-PCI TIMI flow 2, n (%)	5 (45.4%)	4 (44.4%)	6 (60%)	0.740
5	Post-PCI TIMI flow 0, n (%)	2 (18.1%)	0	0	0.835
6	Post-PCI TIMI flow 1, n (%)	0	1 (11.1%)	3 (30%)	0.380
7	Post-PCI TIMI flow 2, n (%)	4 (36.3%)	2 (22.2%)	2 (20%)	0.654
8	Post-PCI TIMI flow 3, n (%)	5 (45.4%)	6 (66.6%)	5 (50%)	0.618
9	Post-PCI TIMI flow 2 and 3, n (%)	9 (81.8%)	8 (88.8%)	7 (70%)	0.579
10	Post-PCI TIMI flow 2 and 3, n (%)	17 (85.0%)		7 (70%)	0.332
11	Pre-PCI TMPG 0/1, n (%)	5 (45.4%)	4 (44.4%)	6 (60%)	0.740
12	Pre-PCI TMPG 2/3, n (%)	6 (54.5%)	5 (55.5%)	4 (40%)	0.740
13	Post-PCI TMPG 0/1, n (%)	3 (27.2%)	1 (11.1%)	3 (30%)	0.578
14	Post-PCI TMPG 2/3, n (%)	8 (72.7%)	8 (88.8%)	7 (70%)	0.578
15	Mean LVEF at presentation (%)	33.18±7.50	33.33±9.35	35.5±8.31	0.786^Ɨ^
16	Death at 30 days, n (%)	5 (45.4%)	2 (22.2%)	8 (80%)	0.039*
17	Death at 30 days, n (%)	7 (35%)		8 (80%)	0.020*

The majority (66.6%, n=20) of our patients had IABP initiation before PCI, i.e. 36.6% (n=11) before coronary angiography and nine patients (30%) between angiography and PCI whereas 10 patients (33.3%) had IABP initiation immediately after PCI.

Effect of Timing of IABP initiation on Mortality

**Figure 1:**
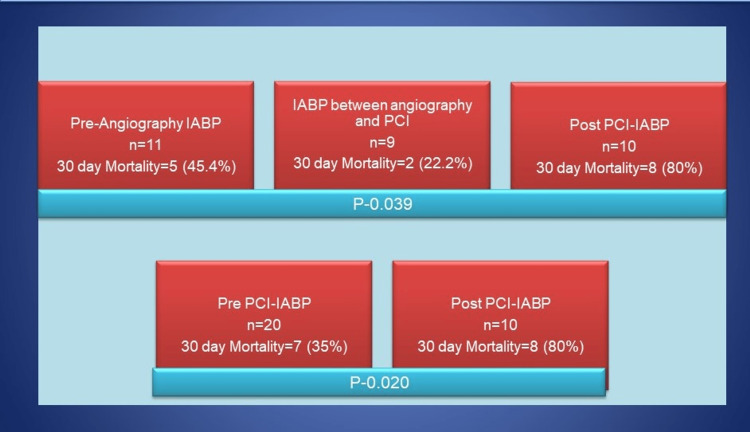
Outcomes on the basis of timing of IABP initiation. Lowest mortality rate was seen when IABP was initiated between angiography and PCI. IABP, intra-aortic balloon pump; PCI, percutaneous coronary intervention.

Pre-PCI vs. post-PCI IABP initiation: On evaluating the effect of timing of IABP initiation on death due to any cause within 30 days of follow-up, we observed that out of 20 patients with pre-PCI IABP initiation, 7 (35%) died compared to 8 (80%) deaths among those who underwent IABP initiation after PCI (n=10) (Figure 1). The difference was statistically significant (relative risk: 0.44; 95% confidence interval: 0.22-0.86; p=0.020).

"Pre-angiography" vs. "post-angiography pre-PCI" vs. "post-PCI" IABP initiation: On further splitting the timing of IABP initiation as pre-angiography, post-angiography but pre-PCI and post-PCI, we observed a 30-day mortality rate of 45.4% (5 out of 11), 22.2% (2 out of 9), and 80% (8 out of 10), respectively. The difference was statistically significant (p=0.039), which suggests that the maximum survival benefit of IABP is harvested when placed after angiography but before PCI. 

In summary, our results suggest that in patients of STEMI with high thrombus burden, better short-term survival is associated with improved TIMI flow rates, improvement in LVEF, and pre-PCI initiation of IABP. Further subgroup analysis, although limited by a small number, revealed that post-angiography but pre-PCI IABP initiation is best in terms of improved survival.

## Discussion

**Figure 2:**
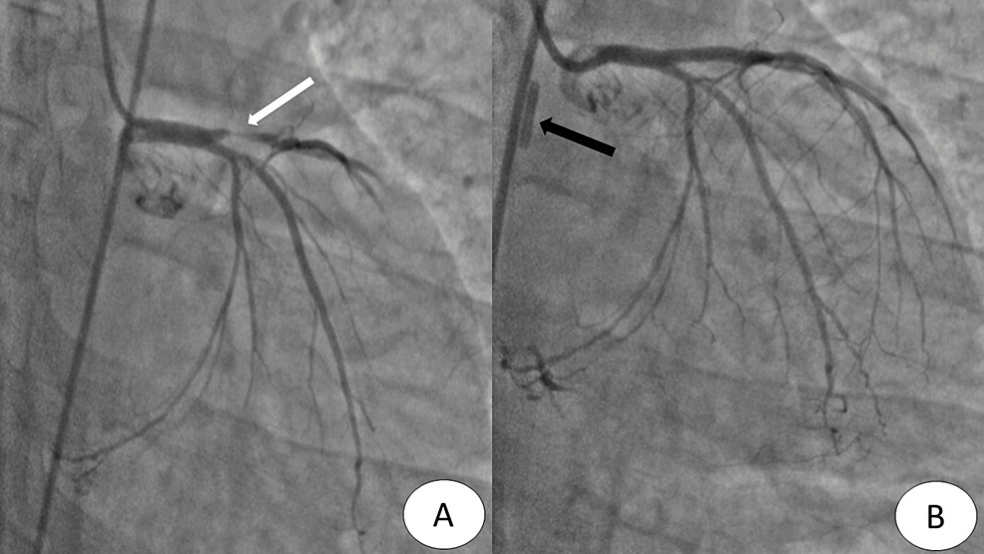
Coronary angiography of one of the patients included in the study. (A) Proximal left anterior descending artery showing Grade 4 thrombus (white arrow). (B) TIMI 3 flow in the vessel after IABP (black arrow) assisted PCI. TIMI, thrombolysis in myocardial infarction; IABP, intra-aortic balloon pump; PCI, percutaneous coronary intervention.

In our study population, 83.3% were male, possibly due to a higher incidence of CAD, the presence of comorbidities, and selection bias created by relatively higher thrombus burden in coronaries of males compared to females [18]. 63.6% of patients had anterior wall myocardial infarction and 93.3% had a cardiogenic shock. The overall 30-day mortality rate of 50% (n=15) seen in our study is similar to that seen (51.6-53.5%) in other studies from Asia [19,20]. A mortality rate of 30% seen in our patients with successful PCI (TIMI 2/3 flow) is significantly lower than 54.8% seen in a similar group of patients in a study by Hawranek M et al. [21] in which 1,116 patients of STEMI as well as NSTEMI underwent IABP initiation along with PCI; however, it included 17.7% patients with resuscitated cardiac arrest. Better results with successful PCI in our study were possibly because none of the patients in our study suffered cardiac arrest prior to PCI. Figure 2 demonstrates a coronary angiographic image with a grade 4 thrombus of one of the patients included in the study who underwent IABP-assisted PCI.

Impact of timing of IABP initiation on TIMI flow, cTFC, and TMPG

Effect of Pre-angiography IABP Initiation on TIMI Flow

Some studies have shown the beneficial impact of IABP on infarct-related artery recanalization [22]. However, no significant benefit of pre-angiography IABP initiation on culprit vessel flow was observed in our study. Contrary to this, a study by Li Z et al. in STEMI patients showed that IABP initiation before angiography had significantly better TIMI flow rates compared to controls [23]. The lack of statistically significant results in our study may be due to the inclusion of patients with cardiogenic shock, delayed presentation, high thrombus load, and possibly a small sample size.

Effect of Pre-PCI vs. Post-PCI IABP Initiation on TIMI Flow, cTIMI Frame Count, and TMPG

In patients with high thrombus burden, a timely support with IABP may potentially improve coronary flow after PCI [24]. However, we observed no significant improvement in cTFC (p=0.428) and TMPG (p=0.33) with pre-PCI IABP initiation. Some other studies have shown no significant beneficial impact of pre- or post-PCI IABP initiation on post-PCI TIMI flow rates in high-risk patients without and with cardiogenic shock, respectively [25,26]. However, a study by Lin Yuan et al. in 218 patients with STEMI and cardiogenic shock undergoing primary PCI showed a significant improvement in TMPG grades 2/3 with pre-PCI IABP initiation compared to post-PCI IABP initiation [27].

Impact of Timing of IABP Initiation on Mortality

We observed that on 30-day follow-up after PCI, patients who survived (n=15) had a significantly higher frequency of pre-PCI IABP initiation (86.6%, n=13) as compared to those who died (46.6%, n=7) (p=0.020). The maximal benefit was observed with post-angiography but pre-PCI IABP initiation (p=0.039), but this observation is limited by a relatively small number of patients. Our results are supported by a randomized controlled trial by Zhou M et al. in patients with acute myocardial infarction and cardiogenic shock undergoing high-risk PCI, wherein early IABP initiation resulted in a significantly reduced perioperative mortality rate and better weaning off the ventilator (p<0.05) [28]. However, our study is different in including only STEMI patients with high thrombus burden. Contrary to this, a meta-analysis by Cui K et al. found no significant difference in 30-day mortality among patients undergoing primary PCI with pre-PCI versus post-PCI IABP initiation with a mortality rate of approximately 36% in both arms (p=0.67) [29]. All patients in this meta-analysis had STEMI with cardiogenic shock.

Similarly, an observational retrospective study on 139 patients with STEMI and cardiogenic shock showed no survival benefit of pre-PCI compared to post-PCI IABP initiation; however, thrombus burden was not assessed in this retrospective analysis [25]. Another retrospective study in 2010 by Abdel-Wahab M et al. [30] on patients with acute coronary syndrome associated with cardiogenic shock showed an in-hospital mortality rate of 19% in the pre-PCI IABP group as compared to 59% in the post-PCI IABP group suggesting that pre-PCI IABP can improve outcomes as observed in our study.

Limitations

This is an observational study with a small sample size with short-term follow-up. Since most patients included in this study were STEMI patients with high thrombus load complicated by cardiogenic shock, the study results cannot be extrapolated to all STEMI patients. There are significant confounding variables like the timing of presentation, the standard of medical and critical care provided, procedural variables, and technical expertise for high-risk intervention. Significant referral bias is possible leading to relatively high mortality as our center is one of the few tertiary care centers of a developing nation where most of the critical patients are referred from peripheral centers.

## Conclusions

Acute ST-elevation MI complicated by cardiogenic shock with high-grade angiographic thrombus has a very high risk for in-hospital death even with PCI. Apart from standard medical therapies and revascularization, IABP used before PCI improves short-term mortality in such patients. Placing an IABP after PCI does not confer any advantage. The best time to place an IABP in achieving good clinical outcomes is before PCI, preferably after diagnostic coronary angiography which also reveals a high-grade thrombus. However, a large randomized controlled study with sufficient sample size and a long-term follow-up is required to strengthen our observations.
